# Animal Welfare Assessment Protocol for Does, Bucks, and Kit Rabbits Reared for Production

**DOI:** 10.3389/fvets.2020.00445

**Published:** 2020-08-07

**Authors:** Antoni Dalmau, Xenia Moles, Joaquim Pallisera

**Affiliations:** Animal Welfare Program, Institute of Agrifood Research and Technology, Girona, Spain

**Keywords:** animal-based parameters, animal welfare, assessment protocol, behavior, health, housing, injuries, rabbits

## Abstract

Rabbits are the third species in terms of number of animals reared for meat production in the world. However, in comparison to other species, very few studies have focused on their welfare. The aim of the present study is to implement an animal welfare-assessment protocol developed through a multidimensional approach and containing a number of animal-based measures for bucks, does, and kit rabbits. Thirty Spanish farms with conventional cages in the first year of integration in an animal welfare certification scheme were visited during 2019 and audited by the same auditor. The protocol is divided into four principles and 11 criteria. The Good Feeding principle includes eight parameters (one animal-based), Good Housing includes 15 parameters (six animal-based), Good Health includes 26 parameters (16 animal-based), and Appropriate Behavior contains nine parameters (four animal-based). In general, the main problems found were the absence of platforms, low space allowance and low height of the cage, inappropriate systems for performing emergency killing, insufficient protection of does from other adjacent does when housed individually, and absence of enrichment material. To a minor degree, but also found in an important number of farms, was a lack of temperature data records, high replacement rates of does, and lack of mortality-rate data records. However, in general, most of the farms obtained a good overall score, the maximum found being 73 out of 100 points. Nevertheless, none of the farms reached an excellent score, and four farms were scored below the 55 points required in the animal welfare certification scheme. The Good Feeding principle obtained the highest score, reaching excellent in all farms, and Appropriate Behavior the lowest one, with values ranging from 21 to 41 points out of 100. The results probably show how, for years, rabbit producers have been very focused on feeding needs and very little attention has been paid to behavioral needs.

## Introduction

Domestic rabbits *(Oryctolagus cuniculus)* bred for meat production represent <1% of the meat produced in the world. However, although in terms of kg, rabbits are negligible in comparison to other species, animal welfare considers individuals and not tons of meat. According to the FAOSTAT, in 2018, the number of slaughtered animals in the world for meat production was 68.785 billion chickens, 1.484 billion pigs, 922 million rabbits, 656 million turkeys, 573 million sheep, 479 million goats, 302 million cattle, and 5 million horses (1). Therefore, rabbits are the third species in terms of number of animals reared for meat production in the world. As happens with other species, the production of these animals has been increasing in the last few years. From 2008 to 2018, the number of rabbits reared in the world for production increased 9.8% (1).

According to Broom (2), the welfare of an animal is its state as regards its attempts to cope with its environment, and stress response plays a key role in these attempts. In fact, stress has been defined as a state that occurs when an animal is required to make abnormal or extreme adjustments, in either its physiology or behavior, in order to cope with adverse aspects of its environment and management (3). Stress affects the animals in different ways, such as reduced feed intake (4, 5), increased disease susceptibility (6), reproductive efficiency (7, 8), or changing the behavior of animals (9, 10).

Probably due to the low economic impact of the production of rabbits worldwide or due to the geographical distribution of this production system, rabbits are by far the meat production species least studied in terms of animal welfare, particularly when compared to pigs, chickens, turkeys, cattle, sheep, and goats (11). For instance, the European Union (EU) funded from 2004 to 2009 one of the most ambitious projects on animal welfare ever developed, the Welfare Quality project. One of the aims of this project was to develop protocols to assess animal welfare in an objective, science-based, and practically applicable way, focusing the assessment on animal-based parameters (12). However, this project was focused only on cattle, pigs, and chickens. After this project, and following the principles stated by the Welfare Quality project, the EU funded a second one, named AWIN, which covered the omitted species in the previous one: turkeys, sheep, goats, and horses. Yet, rabbits were never considered in either of the two European projects. Nonetheless, rabbit producers are subjected to the same challenges as other producers: a higher demand from consumers for animal-friendly production systems and a greater production efficiency to increase marginal benefits. In both cases, a better knowledge of animal welfare and tools for their assessment plays a central role. These tools, or animal welfare-assessment protocols, can be used by farmers to identify critical points in the farm for investments, to compare their own results with those from other producers to perform self-assessments, and to create communication channels with the consumer to give an added value to the farms with better conditions.

De Jong et al. (13) presented a first step in the development of an animal welfare-assessment protocol for commercially housed rabbits. This consisted of describing possible parameters for the different criteria and principles as used in Welfare Quality project. This was done by combining the little information existing in the literature and the opinion of experts from different countries. This basis was tested in Spain to build a possible animal welfare protocol for rabbits and, from 2016 to 2018, it was tested in different rabbit farms for meat and fur production. According to the results obtained, some extra parameters were added, and others changed. Then the thresholds for the different measures and a score system were developed. The objective of the present study was to present the protocol based on the Welfare Quality approach for discussion after its implementation in 30 farms assessed in Spain that were interested in achieving certification on animal welfare for does, bucks, and kit rabbits.

## Methods

Thirty Spanish rabbit farms with conventional cages were visited from March to December 2019 and assessed by means of an animal welfare protocol for does, bucks, and kit rabbits in a single visit. These farms were audited within an animal welfare certification system (Welfair™; https://www.animalwelfair.com/) and assessed in all cases by the same auditor, who was trained according to the training procedure established in Welfare Quality (12). The system was presented to different national congresses and meetings and farmers volunteered to be audited as part of the study. In all cases, they had the opportunity to check and test the protocol for doing a self-assessment in their farms before the audit, and they were aware that 55 points out of 100 was the cut off for these protocols. However, it is unknown how many of them carried out this self-assessment, whether they used a part or all the measures of the protocol for this purpose, or if they just did an overview to the protocol before confirming their interest in being audited. In any case, the results presented in this study were collected during the first assessment done by an external auditor in all 30 farms assessed. Cages to be assessed were selected randomly throughout the buildings to be representative of the overall picture of the farm. Although the protocols can be used for bucks and does, only one of the assessed farms had males for reproduction, and in the rest, females were artificially inseminated. The Welfare Quality schema provides four different principles to assess animal welfare, and these are divided into 12 criteria (Table 1). This is the structure used in the present study. However, as in the present protocol it was not possible to identify any good parameter to assess the positive emotional state criterion, the protocol includes only 11 criteria. Globally, the Good Feeding principle includes eight parameters (one animal-based); Good Housing includes 15 parameters (six animal-based); Good Health includes 26 parameters (16 animal-based); and Appropriate Behavior contains nine parameters (four animal-based).

**Table 1:** Principles and criteria defined in the European Welfare Quality project to assess animal welfare (12).

**Principles**	**Criteria**
Good Feeding	Absence of prolonged hunger
	Absence of prolonged thirst
Good Housing	Comfort around resting
	Thermal comfort
	Ease of movement
Good Health	Absence of injuries
	Absence of diseases
	Absence of pain induced by management
Appropriate Behavior	Social behavior
	Other behaviors
	Human-animal relationship
	Positive emotional state

### Good Feeding

The Good Feeding principle is assessed by means of the combination of two criteria: absence of prolonged hunger (65% of the total score) and absence of prolonged thirst (35% of the total score; Table 2). In relation to the first criterion, the protocol states that, for the body-condition parameter, a total of 24 bucks and 34 does are assessed (when possible, 17 around mounting or insemination and 17 just before weaning). If there are no males in the farm, a total of 50 does are assessed (when possible, 25 around insemination and 25 just before weaning). Presence of bad body condition (thinness) is assessed visually, considering an animal to be lean when hips and backbone are very prominent. The cleanliness of the feeders, number of drinking points per doe/buck, functioning of the drinkers, and cleanliness of the drinkers parameters are assessed in 24 cages for bucks and 51 for does (when possible, 17 in the first week post-partum, 17 around insemination, and 17 post-weaning). If the farm does not contain bucks, it is assessed in 75 cages for does (when possible, 25 of each type). A feeder is considered dirty when it contains corrupted food, compacted dry food, and mold. A bad functioning of the drinkers is considered when there is an insufficient flow or if they are dripping. Access to milk in kits is assessed by asking the farmer and, if possible, checking it during the visit, if all of the kits during the first 7 days of life are checked after the visit of the doe to the nest to ensure that all of them have had milk. Access to food by kits and height of drinkers are only assessed if kits of at least 21 days of age are present (Table 2). If they do not have access to solid food, 20 points are subtracted from the whole absence of prolonged hunger criterion. If the drinker for these kits is not <22 cm from the floor, 20 points are subtracted from the absence of prolonged thirst criterion.

**Table 2:** Parameters used to assess the criteria of absence of prolonged hunger and absence of prolonged thirst.

**Criterion**	**Parameter**	**Weight (%)**	**Categories**	**Definition of categories**	**Score**
Absence of prolonged hunger	Body condition	70	Excellent	0% of lean animals	100
			Acceptable	Up to 3% of lean animals	70
	Cleanliness of feeders	15	Excellent	100% of clean feeders	100
			Acceptable	At least 97% of clean feeders	45
	Access to milk in kits	15	Excellent	All kits checked after doe's visit the first week	100
			Not acceptable	Any kit not checked after doe's visit	0
	Access to food in kits	0	Acceptable	Solid food for kits of 21 days or older	0
		−20	Not acceptable	No solid food for any kit of 21 days or older	−20
Absence of prolonged thirst	Drinking points per doe/buck	45	Excellent	More than 1 drinking point per doe/buck	100
			Acceptable	A ratio of 1 drinking point for 1 doe/buck of fresh water tested and working	65
	Functioning of drinkers	35	Excellent	100% with a good water flow	100
			Acceptable	At least 97% with a good water flow	55
	Cleanliness of drinkers	25	Excellent	100% of clean drinkers	100
			Acceptable	At least 97% of clean drinkers	50
	Height of drinkers	0	Excellent	Drinker for kits at 13 cm from the floor or less	0
		−20	Acceptable	Drinker for kits at >13 and <23 cm from floor	−10
			Not Acceptable	Any other situation for kits older than 21 days	−20

*The weight means which percentage of the score of the total criterion is represented by each parameter. Each parameter can be assessed according to different categories (excellent, acceptable, and not acceptable) and are scored accordingly*.

### Good Housing

The Good Housing principle is assessed by means of the combination of three criteria: comfort around resting (40%), thermal comfort (25%), and ease of movement (35% of the total score; Table 3). The wet animals, dirty animals, presence of resting mats, height of the cage/pen, and stocking density parameters are assessed in 24 cages for bucks and 51 for does (when possible, 17 in the first week post-partum, 17 around insemination, and 17 post-weaning). If the farm does not contain bucks, it is assessed in 75 cages for does (when possible, 25 of each type). A wet animal is considered when any part of the fur is wet. For dirtiness, two categories are considered. The animal is scored as moderately dirty when from 10 to 30% of the body is dirty, and severely dirty when more than 30% of the body is dirty. The stocking density is assessed in cm^2^ of free space per animal. The parameters of free movement, panting, and shivering are assessed in a total of 10 bucks and 40 does not assessed for other parameters (if there are not bucks in the farm, 50 does are assessed). Each animal is assessed during a time of 2 min. Free movement is defined as the capacity of the animal for performing hopping, jumping, and turning. An animal is considered as panting when it is breathing with short and quick breaths and with the mouth open. The fully stretched lying animals parameter is assessed in all animals assessed for dirtiness and free movement, a total of 34 bucks and 91 does, or 125 does if there are no bucks in the farm. The quality littered floor parameter is assessed only in cages with does and kits in the first week after kindling (from 17 to 25 cages) and refers to the material present in the nest. The presence of an elevated platform parameter is assessed only in cages with kits older than 21 days (from 17 to 25 cages). The dust, light quality, environmental temperatures, and burning hair parameters are assessed globally in the facilities of each farm. The dust parameter is assessed by means of a black surface of ~10 ^*^ 15 cm (DINA 4-folds in four pieces) and left during the assessment at the center of the building housing the rabbits at the same height as their heads. At the end of the visit, the level of dust accumulated is assessed considering three possibilities: no evidence of dust, minimal evidence of dust (a thin covering of dust), and a lot of dust (possible to write on the paper with a finger, or paper not visible). The quality of light is considered correct when it is possible to check all of the animals and if at least 8 h of light and darkness are provided. When it is considered unacceptable, 20 points are subtracted from the comfort around resting criterion. The temperature parameter is assessed according to the temperature data record in the farm. If there are no data, 0 points are given. If there are data, excellent is given when the temperatures, maximum and minimum, range from 1 to 28°C, respectively. Acceptable is considered when, up to 2 days in the last 3 months, the temperature registered is out of this range and, finally, unacceptable in any other case. The burning hair parameter is related to the burning of molted hair accumulated in the cages for improving environmental conditions. In this case, if the temperature is outside of the proposed ranges (1–28°C) during this practice or not registered, 20 points are subtracted from the thermal comfort criterion.

**Table 3:** Parameters used to assess the criteria of comfort around resting, thermal comfort, and ease of movement.

**Criterion**	**Parameter**	**Weight (%)**	**Categories**	**Definition of categories**	**Score**
Comfort around resting	Fully stretched animals	15	Excellent	At least 20% of the animals fully stretched	100
			Acceptable	At least 10% of the animals fully stretched	45
	Wet animals	20	Excellent	<5% of wet animals	100
			Acceptable	<10% of wet animals	60
	Dirty animals	20	Excellent	Up to 2% moderately and 0% severely dirty	100
			Acceptable	Up to 4% moderately and 2% severely dirty	60
	Dust	15	Excellent	No dust presence	100
			Acceptable	Minimal dust present	70
	Presence of Resting mat	30	Excellent	100% of does and bucks with resting mat	100
			Acceptable	At least 50% of does and bucks with resting mat	50
	Presence of an elevated platform	0	Excellent	100% of the cages with a platform	0
		−20	Acceptable	At least 50% of the cages with a platform	−10
			Not acceptable	Any other situation	−20
	Light quality	0	Acceptable	8 h of light and 8 h of darkness and enough light to check animals	0
		−20	Not acceptable	Any other situation	−20
	Quality of littered floor	0	Excellent	Clean and dry litter in all nests	0
		−20	Acceptable	No clean or dry litter in up to 2 nests	−10
			Not acceptable	Any other situation	−20
Thermal comfort	Temperature	100	Excellent	Last 3 months with range of 1–28°C	100
			Acceptable	Up to 2 days out of this range	50
	Burning hair	0	Acceptable	During burning hair not >28°C	0
		−20	Not acceptable	No data or >28°C	−20
	Panting	0	Excellent	0% of animals panting	0
		−100	Acceptable	Up to 4% of animals panting	−50
			Not acceptable	More than 4% of animals panting	−100
	Shivering	0	Excellent	0% of animals shivering	0
		−100	Acceptable	Up to 4% of animals shivering	−50
			Not acceptable	More than 4% of animals shivering	−100
Ease of movement	Free movement	30	Excellent	100% of the animals with free movement	100
			Acceptable	At least 97% of the animals	65
	Height of the cage	30	Excellent	38 cm at least in 90% of the cages	100
			Acceptable	32 cm at least in 90% of the cages	50
	Stocking density	40	Excellent	At least 3,500 cm^2^ per doe/buck in 90% of cages	100
			Acceptable	At least 2,500 cm^2^ per doe/buck in 90% of cages	60

*The weight means which percentage of the score of the total criterion is represented by each parameter. Some parameters, such as light quality, are only considered in the score when the acceptable value is not achieved, subtracting points from the overall score. Each parameter can be assessed according to different categories and are scored accordingly*.

### Good Health

The Good Health principle is assessed by means of the combination of three criteria: absence of injuries (40%), absence of diseases (40%), and absence of pain induced by management (20%, Table 4). The parameters of wounds on the body, wounds on the ears, fallen ears, pododermatitis, gait score, nasal discharge, ocular discharge, dermatophytosis/dermatitis/abscesses, neck torsions, enteropathy, diarrhea, mange, risk of injuries, and cleanliness of the housing system are assessed in 24 cages for bucks and 51 for does (when possible, 17 in the first week post-partum, 17 around insemination, and 17 post-weaning). If the farm does not contain bucks, it is assessed in 75 cages for does (when possible, 25 of each type). For wounds on the body, a lesion is considered a fresh scratch or open lesion equal or bigger than 2 cm in any part of the animal and not healed. Any animal with these lesions is assessed as moderately injured. Nevertheless, in case of a lesion of equal or more than 5 cm, the animal is assessed as severely injured. For wounds on the ears, no distinction for size is made, and only lesions of equal or more than 2 cm are considered. However, in this case, old lesions are distinguished from fresh lesions. Fallen ears are considered just as the absence or presence of the problem, and only the worse of the two ears is considered. This parameter is not considered for certain breeds with fallen ears, such as beliers. Pododermatitis considers three cases: no problem, when the feet are fine; moderate problems, if there is no hair, with a callus formed, and the area affected being longer than 2 cm; and severe problems, if there is an open lesion. Gait score considers three possibilities: no problems, if the animal does not have any difficulty in moving; moderate problem, if the animal has any difficulty in moving; and severe problem, if the animal has several difficulties (no use of one leg or minimum weight bearing). Nasal and ocular discharge is only considered as the presence or absence of the problem. Signs of conjunctivitis are considered as the presence of ocular discharge. Any sign of skin inflammation is considered, as the presence or absence, in the parameter of dermatophytosis/dermatitis/abscesses. For neck torsion, three conditions are considered: absence, when the neck is perfect; moderate problem, when the animal has a neck torsion but is able to eat and drink with no difficulties; and severe problem, when the neck torsion makes access to food and water difficult for the animal. Enteropathy is assessed by palpation of the abdomen and is considered present when this is hard. Diarrhea is assessed as the presence of liquid feces around the anus of the animal. Mange is assessed as its presence or absence. Risk of injuries is assessing the risk for the animals to be injured by bad maintenance of the cages or other elements in their surroundings. This parameter subtracts up to 30 points from the absence of injuries criterion if more than one cage with a problem is found. The cleanliness of the housing system parameter has three possibilities: the cage is clean; the cage is partly dirty, when only a part of the cage is affected (including a lot of presence of hair, compacted dry food, and mold); and a dirty cage, when the entire cage is very dirty. This parameter subtracts up to 20 points from the absence of diseases criterion. The coughing and sneezing parameters are assessed in a total of 10 bucks and 40 does not assessed for other parameters (if there are no bucks in the farm, 50 does are assessed). Each animal is assessed during a time of 2 min, and the presence or absence of coughing or sneezing during this period is considered. Hairless areas are assessed only in bucks (in a total of 24 animals) and considered present when there is an area of equal or more than 2 cm without hair. Mortality and culling rates are assessed according to the records of the farm in the last 3 months. Mortality considers only adult does and bucks deaths in the farm and not culled by the farmer. The parameters of replacement, time between parturitions, age of weaning, mutilations used for identification, and procedure for emergency killing are asked of the farmer and, when possible, assessed during the visit. The presence of flies parameter is assessed by observing the facilities where animals are housed, and three possibilities are considered: no problem, when no presence of flies or fly eggs is observed overall in the farm; moderate problems, when the presence of flies or fly eggs is observed (which indicates a problem with flies only in the past [eggs] or only in the present [flies]); and severe problem, when the presence of flies and fly eggs is observed (which indicates a chronic problem of flies in the farm).

**Table 4:** Parameters used to assess the criteria of absence of injuries, absence of diseases, and pain induced by management procedures.

**Criterion**	**Parameter**	**Weight (%)**	**Categories**	**Definition of categories**	**Score**
Absence of injuries	Wounds on the body	25	Excellent	Up to 2% moderate and 0% severe	100
			Acceptable	Up to 4% moderate and 2% severe	60
	Wounds on the ears	15	Excellent	Up to 2% old and 0% fresh lesions	100
			Acceptable	Up to 4% old and 2% fresh lesions	45
	Fallen ears	10	Excellent	Up to 2% with fallen ears	100
			Acceptable	Up to 4% with fallen ears	70
	Pododermatitis	30	Excellent	Up to 50% moderate and 5% severe	100
			Acceptable	Up to 65% moderate and 8% severe	50
	Gait score	20	Excellent	Up to 2% moderate and 0% severe	100
			Acceptable	Up to 4% moderate and 2% severe	50
	Hairless areas	0	Excellent	0% of animals affected	0
		−10	Acceptable	Up to 13% of animals affected	−5
			Not acceptable	More than 13% of animals affected	−10
	Risk of injuries	0	Acceptable	No cages with risk of injuries	0
		−30	Not accept	1 cage with risk of injuries	−15
			Not accept	More than 1 cage with risk of injuries	−30
Absence of diseases	Mortality	10	Excellent	Up to 3% in the last 3 months	100
			Acceptable	Up to 5% in the last 3 months	70
	Culling	5	Excellent	At least equal to or higher than mortalities	100
			Acceptable	At least 50% of mortality rates	40
	Replacement	5	Excellent	<80% of females per year	100
			Acceptable	<110% of females per year	40
	Time between parturitions	5	Excellent	At least 49 days between parturitions	100
			Acceptable	At least 42 days between parturitions	40
	Coughing	10	Excellent	0% of animals coughing during 2 min	100
			Acceptable	Up to 3% of animals coughing	70
	Sneezing	10	Excellent	0% of animals sneezing during 2 min	100
			Acceptable	Up to 3% of animals sneezing	70
	Nasal discharge	7	Excellent	Up to 2% of animals affected	100
			Acceptable	Up to 4% of animals affected	40
	Ocular discharge	8	Excellent	Up to 2% of animals affected	100
			Acceptable	Up to 4% of animals affected	50
	Dermatophytosis, dermatitis, abscesses	10	Excellent	0% of dermatophytosis and up to 4% of animals with dermatitis or abscesses	100
			Acceptable	0% of animals with dermatophytosis and up to 10% of animals with dermatitis or abscesses	70
	Neck torsions	10	Excellent	Up to 2% moderate, 0% severe	100
			Acceptable	Up to 4% moderate, 2% severe	70
	Enteropathy	10	Excellent	0% of animals affected	100
			Acceptable	Up to 2% of animals affected	70
	Diarrhea	10	Excellent	0% of animals affected	100
			Acceptable	Up to 4% of animals affected	70
	Mange	0	Acceptable	0% of animals affected	0
		−40	Not acceptable	At least one animal affected	−40
	Cleanliness of facilities	0	Acceptable	Up to 2 cages partly dirty	0
		−20	Not acceptable	Up to 5 cages partly dirty and up to 2 cages dirty	−10
			Not acceptable	Any other case	−20
	Age of weaning	0	Acceptable	35 days old or older	0
		−20	Not acceptable	Before to 35 days old	−20
	Flies presence	0	Acceptable	No flies neither eggs of flies present	0
		−20	Not acceptable	Flies or eggs of flies present	−10
			Not acceptable	Flies and eggs of flies present	−20
Absence of pain induced by management	Killing methods	100	Excellent	Penetrative captive bolt with pithing, penetrative captive bolt with bleeding or penetrative captive bolt with neck dislocation Electronarcosis with neck dislocation or electronarcosis with bleeding Lethal injection	100
			Not acceptable	None of the previous systems	0
	Mutilations for identification	0	Excellent	No mutilations performed	0
		−20	Not acceptable	Mutilations for identification in any part of the body	−20

*The weight means which percentage of the score of the total criterion is represented by each parameter. Some parameters, such as risk of injuries, are only considered in the score when the acceptable value is not achieved, subtracting points from the overall score. Each parameter can be assessed according to different categories and are scored accordingly. In some cases, the category not acceptable can have more than one score*.

### Appropriate Behavior

The Appropriate Behavior principle is assessed by means of the combination of three criteria: social behavior (35%), other behaviors (35%), and human-animal relationship (30%, Table 5). The parameters of negative social behavior, abnormal behaviors, and human approach test are assessed in a total of 10 bucks and 40 does not assessed for other parameters (if there are no bucks in the farm, 50 does are assessed). Negative social behavior is considered as any such event in which a doe or a buck is biting another one, including kits in the same cage or any animal in other adjoining cages for a time of 2 min. Abnormal behavior consists of animals scratching or biting the cage or performing repetitive behaviors without an apparent objective (stereotypies) and it is assessed for a time of 2 min per cage. The human approach test is performed after the 2 min dedicated to assess the other behavioral measures. For 30 s, the assessor would be in front of the cage of the animal touching the frontal area of the cage with a short stick (no more than 10 cm long, a new stick is used for each animal). Three possibilities are considered: confident, if the animal touches or sniffs the stick; interested, if the animal shows some interest in the stick and approaches to at least 10 cm from the stick; not interested or fearful, any other situation. The isolated animals and presence of enrichment material parameters are assessed in a total of 34 bucks and 91 does (or 125 does is there are no bucks in the farm). Isolated animals are positively considered when adult animals, due to the distance between cages or to the provision of solid separations, are protected from physical contact from other adults. In addition, it is negatively considered if any animal is visually isolated from other animals. The presence of enrichment material, different to metal cans, such as cubes of dried hay or wood sticks, is considered as being present or absent. The availability of nesting material parameter will be assessed, when possible, in 10 does 24 h prior to the date expected for kindling by checking for the provision of enough dry and clean nesting material. The parameters of training of personnel, time of having access to the nest, and touching kits every day are assessed by asking the farmer, although the last two parameters can be checked as well during the audit. Training of personnel considers three levels: all personnel in the farm in contact with the animals are trained in animal welfare; at least one person is trained in animal welfare; and none of the persons is trained in animal welfare. Certificates for any training must be shown. Time of accessing the nest assesses the regularity in giving the doe access every day to the nest at the same hour and considers three categories: no problem, when <1 h of difference is found among different days; moderate problems, when a difference among days of more than 1 h but <2 h is found; and severe problems, when the difference is of more than 2 h. Touching the kits every day assesses whether the manager is touching the kits for the first week of age at least once a day.

**Table 5:** Parameters used to assess the criteria of social behavior, other behaviors and human-animal relationship.

**Criterion**	**Parameter**	**Weight (%)**	**Categories**	**Definition of categories**	**Score**
Social behavior	Negative social behavior	100	Excellent	No animals biting other animals	100
			Not acceptable	One animal biting another animal	0
	Isolated animals	0	Excellent	No does/bucks visually isolated, but all of them physically isolated from other adults	0
		−100	Not acceptable	Up to 5% of does/bucks visually isolated and at least 50% physically isolated	−50
			Not acceptable	Any other situation	−100
Other behaviors	Abnormal behavior	60	Excellent	0% with abnormal behavior	100
			Acceptable	Up to 4% of animals affected	55
	Enrichment material	40	Excellent	100% with enrichment material	100
			Acceptable	At least 50% with enrichment material	60
	Nesting material	0	Excellent	Nesting material in 100% of the cases	0
		−20	Acceptable	Up to 1 doe without nesting material	−10
			Not acceptable	Any other case	−20
	Time of access to the nest	0	Excellent	<1 h of difference day to day	0
		−20	Acceptable	>1 and <2 h of difference day to day	−10
			Not acceptable	Any other case	−20
Human-animal relationship	Human approach test	70	Excellent	At least 20% touching the stick and 40% not touching the stick but interested	100
			Acceptable	At least 10% touching the stick and 20% not touching the stick but interested	50
	Training of personnel	30	Excellent	All personnel in contact with animals trained in animal welfare	100
			Acceptable	At least one person trained in animal welfare	50
	Touching the kits	0	Excellent	All kits in the nests are touched once a day	0
		−10	Not acceptable	Not all kits are touched	−10

*The weight means which percentage of the score of the total criterion is represented by each parameter. Some parameters, such as isolated animals, are only considered in the score when the acceptable value is not achieved, subtracting points from the overall score. In some cases, the category Not acceptable can have more than one score*.

### Overall Assessment

The Welfare Quality provides a final score for a farm as a result of the combination of the scores of the different principles. When the final score is between 0 and 19 points, the farm is considered not classified. When the final score is between 20 and 54 points, the farm is considered acceptable. When the final score is between 55 and 79 points, the farm is considered enhanced; and, finally, a farm from 80 to 100 points is considered excellent. In the present protocol, the same system was adopted, but changing the category not classified to not acceptable. For an overall assessment of a farm, the four principles of the rabbit protocol have different weights because of the number or importance of the measures included in the specific principles (8, 15, 26, and 9 parameters for principle, respectively). Consequently, to obtain the final score of a farm, 15% depends on the Good Feeding principle, 30% on the Good Housing principle, 35% on the Good Health principle, and 20% on the Appropriate Behavior principle. The overall score can range from 0 to 100 points.

## Results

### Good Feeding

The Good Feeding principle is assessed by means of the combination of two criteria: absence of prolonged hunger and absence of prolonged thirst. Ninety-seven percent of the farms (*n* = 29) obtained 100 points for the absence of prolonged hunger criterion. That means that all does or bucks were found with a good body condition, the feeder was clean, kits were checked once a day to ensure that milk was taken in during the first 7 days of life, and that solid food was provided at least at 21 days of age. In one farm 20% of the feeders were found dirty and was scored with a 0 for this parameter. Consequently, the absence of prolonged hunger criterion obtained 85 points for this farm, and the 30 farms were classified as excellent for the criterion.

In the case of the absence of prolonged thirst criterion, only one farm had more than one drinking point per adult animal, obtaining the 100 points in this parameter. All of the rest of the farms had at least one drinker tested and working properly per animal, so they obtained 65 points. No problems of insufficient flow or dripping were found in any drinker of the 30 farms, so all of them obtained 100 points. Only in one farm was a problem found of dirtiness in the drinkers, reaching 8% of the drinkers assessed (higher than the acceptable level of 3%), so 0 points were given for this parameter to this farm. In relation to the height of the drinkers for the kits after 21 days of age, in 50% of the cases (*n* = 15), the drinker was below 14 cm from the floor, obtaining an excellent. In 37% of the cases (*n* = 11), the drinker was between 14 and 22 cm from the floor and 10 points were subtracted from the final score of the criterion; finally, in 13% (*n* = 4) the height was higher than 22 cm, the maximum being found 27 cm from the floor, and 20 points were subtracted from the whole criterion. Globally, 46% of the farms (*n* = 14) were classified as excellent for this criterion, with scores ranging from 85 to 90 points. The other 64% of the farms (*n* = 16) were classified as enhanced for the criterion, with scores ranging from 65 to 75 points.

When the score of the whole principle was considered, all farms obtained an excellent, with scores ranging from 85 to 97 points.

### Good Housing

The Good Housing principle is assessed by means of the combination of three criteria: comfort around resting, thermal comfort, and ease of movement.

In relation to the comfort around resting criterion, 80% of the farms (*n* = 24) had at least 20% of the animals fully stretched, this percentage ranging from 20 to 65% of the animals. Twenty-seven percent had from 20 to 25% of animals stretched; 17% from 25 to 30%; 27% from 31 to 35%; and the remaining 9% more than 36% of animals fully stretched. In all of these cases, this parameter was scored with 100 points. Ten percent of the farms (*n* = 3) had more than 10% of fully stretched animals and <20%, obtaining 45 points for this parameter. Finally, another 10% of the farms had <10% of animals fully stretched, the minimum percentage observed being 3%. In these cases, the score for the parameter was 0 points. No wet or dirty animals were observed in any of the 30 farms assessed; consequently, 100 points were obtained in all cases for these two parameters. In 90% of the farms (*n* = 27), all of the assessed animals had resting mats in good conditions of maintenance and 100 points were given. In one farm, 63% of the animals had resting mats and 50 points were given. In other two farms <50% of the animals had a resting mat, and 0 points were given. Both dust and quality of littered floor obtained an excellent in all 30 farms assessed. Light quality was excellent in 87% of the farms (*n* = 26), but in the other four farms <8 h of light were provided and, consequently, 20 points were subtracted from the criterion. In addition, none of the 30 farms had an elevated platform for does with kits at 21 days of age or older, so 20 points were subtracted from the criterion for all the farms. Globally, 63% (*n* = 19) of the farms were classified as excellent (just 80 points in all cases); 27% (*n* = 8) of the farms were classified as enhanced (scores ranging from 65 to 72 points); and 10% (*n* = 3) were classified as acceptable (ranging from 35 to 52 points).

In relation to the thermal comfort criterion, none of the animals assessed in any of the farms were found panting or shivering. However, in only 67% of the farms (*n* = 20) was there a data record of at least 3 months of daily maximum and minimum temperatures, so, as the values were within the range of 1–28°C, these farms obtained 100 points and were classified as excellent for the criterion. The rest 33% (*n* = 10) obtained 0 points and were classified as not acceptable for the criterion.

**Figure 1:**
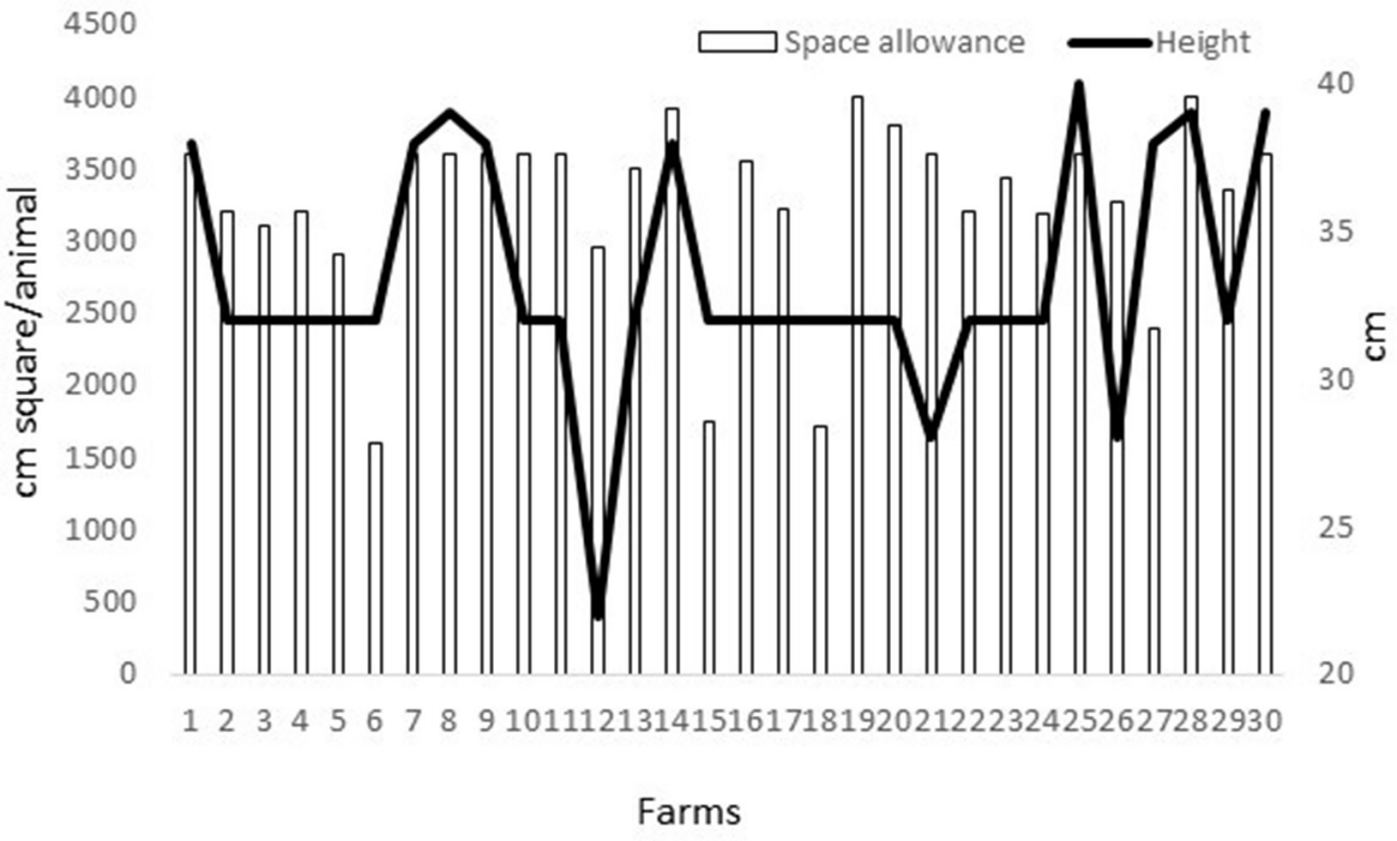
Space allowance (cm^2^/doe or buck; with bars) and height (cm; black line) found in the 30 Spanish farms of bucks, does, and kit rabbits assessed during 2019.

In relation to the ease of movement criterion, 80% of the farms (*n* = 24) had all of the animals with free movement and 100 points were obtained, while the rest 20% (*n* = 6) had <97% of the animals in these conditions, and 0 points were obtained. The height of the cages was of at least 38 cm in 30% of the farms (*n* = 9), the maximum being 40 cm of height (Figure 1). These farms were scored with 100 points. In 60% of the farms (*n* = 18), the height was exactly 32 cm, obtaining 50 points. Finally, in 10% of the farms (*n* = 3) the height was below 32 cm (ranging from 22 to 28 cm), and they were scored with a 0. In 50% of the farms (*n* = 15), at least 3,500 cm^2^ per doe/buck were provided, ranging from 3,500 to 4,000 cm^2^, and were scored with 100 points (Figure 1). In 37% of the farms (*n* = 11) at least 2,500 cm^2^ per adult were given, ranging from 2,900 to 3,450 cm^2^, and were scored with 60 points. Thirteen percent (*n* = 4) provided <2,500 cm^2^ to the animals, ranging from 1,600 to 2,400 cm^2^, and were scored with 0 points. Globally, 50% of the farms (*n* = 15) were classified as excellent for this criterion, with scores ranging from 85 to 100 points. Thirty percent of the farms (*n* = 9) were classified as enhanced, with scores ranging from 55 to 75 points. Thirteen percent of the farms (*n* = 4) were classified as acceptable, with scores ranging from 20 to 45 points. Finally, 7% (*n* = 2) were classified as not acceptable for ease of movement, with a score in both cases of 15 points.

When the score of the Good Housing principle was considered, 43% (*n* = 13) of the farms obtained an excellent (values ranging from 83 to 92 points). Forty-three percent (*n* = 13) of the farms obtained an enhanced (values ranging from 55 to 78 points). Finally, 13% (*n* = 4) of the farms obtained an acceptable (values ranging from 28 to 51 points).

### Good Health

The Good Health principle is assessed by means of the combination of three criteria: absence of injuries, absence of diseases, and absence of pain induced by management.

In relation to the absence of injuries criterion, all farms assessed had 0% of animals with wounds on the ears, fallen ears, or gait score, and the only farm where bucks were present also had 0% of the animals with hairless areas. As a result, the score of excellent (100 points) was obtained for all of these farms in these parameters. In addition, 87% of the farms (*n* = 26) had an excellent for wounds on the body (100 points), two other farms had up to 4% of animals moderately injured (60 points), and the other two had 6 and 12% of animals moderately injured, respectively (0 points). None of the farms had severely injured animals. In the case of pododermatitis, the moderate cases ranged from 10 to 40% of the animals, the severe cases being what determined the score for the farm. In two farms, the percentage of severe cases of pododermatitis was 0% and in 12 other farms the percent was below 5%; consequently, 47% of the farms were scored with an excellent. Twenty-three percent of the farms (*n* = 7) were between 6 and 8% of severe cases of pododermatitis, being scored with 50 points. Finally, 30% of the farms (*n* = 30) had more than 8% of severe cases, the maximum found being 20% of animals affected. In these cases, the farms were scored with 0 points for this parameter. Seventy-three percent of the farms (*n* = 22) had no problems in any of their cages, regarding having a risk of injuries for the animals. However, for the rest of the farms, 27% (*n* = 8), at least two cages with a risk of injuries were found, so 30 points were subtracted from the whole criterion of absence of injuries. Globally, 33% of the farms (*n* = 10) obtained 100 points for this criterion, and 4 other farms obtained at least 85 points, so 47% of the farms were classified as an excellent for comfort around resting to absence of injuries. Forty percent of the farms (*n* = 12) were classified as enhanced (scores ranging from 55 to 70 points). Finally, 13% of the farms (*n* = 4) were classified as acceptable (scores ranging from 40 to 45 points).

**Figure 2:**
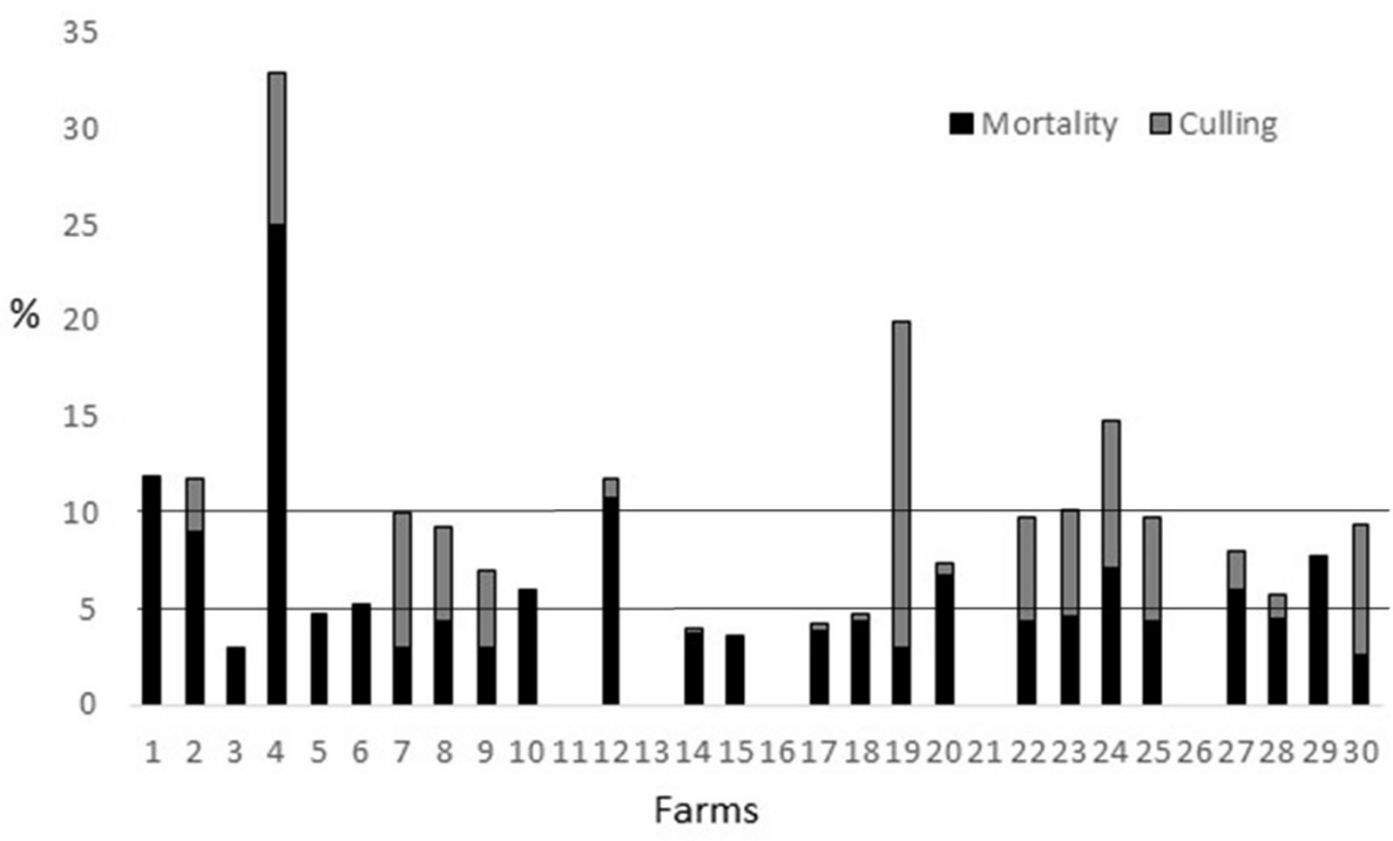
Percentage of mortality (black part of the bar; %) and percentage of culling (gray part of the bar, %) registered during a period of 3 months in the 30 Spanish farms studied during 2019. The absence of a bar (black, gray, or both) means absence of data records for this farm.

In relation to the absence of diseases criterion, the parameters for coughing, sneezing, ocular discharge, neck torsion, and cleanliness of facilities obtained an excellent in all farms assessed, with no problems observed in any animal or cage. Seventeen percent of the farms (*n* = 5) obtained an excellent for mortality, with values ranging from 2.6 to 3%, and 33% of the farms (*n* = 10) obtained 70 points, ranging from 3.5 to 4.8% (Figure 2). Finally, 33% of the farms (*n* = 10) were between 5.2 and 25% of morality and another 17% (*n* = 5) had no data, in both cases being scored with a 0. Thirty percent of the farms (*n* = 9) had equal or higher percentages of animals culled as mortality rates. Thirty-seven percent of the farms (*n* = 11) had culling rates below 50% of mortality rates and another 33% (*n* = 10) had no data on culling rates, so 70% of the farms had a 0 for this parameter (Figure 2). Thirty percent of the farms (*n* = 4) had a replacement percentage of <80% per year, ranging from 35 to 70%, and were scored with 100 points. Another 17% of the farms (*n* = 5) had a replacement percentage of <110% per year, ranging from 90 to 108%, and scored with 40 points. Finally, 70% of the farms (*n* = 21) were scored with 0 points, ranging from 118 to 140% of replacement per year. Thirteen percent of the farms (*n* = 4) had at least 49 days between parturitions, ranging from 49 to 90 days, and scored with 100 points. All the rest of the farms had at least 42 days between parturitions and received 40 points. Eighty-three percent of the farms (*n* = 25) had up to 2% of animals with nasal discharge, being scored with 100 points. In the rest of the farms, 17% (*n* = 5) had more than 4% of animals with nasal discharge, ranging from 8 to 12% of animals affected, and were given 0 points. Ninety-seven percent of the farms (*n* = 29) did not have any problem with dermatophytosis, dermatitis, or abscesses, and they were scored with 100 points, but in one farm 8% of the animals were found with dermatitis, and the farm was given 70 points. Ninety-seven percent of the farms (*n* = 29) did not have any problem with enteropathy, and were scored with 100 points, but in one farm, 8% of the animals were affected, and the farm was scored with 0 points. Forty percent of the farms (*n* = 12) had 0% of animals with diarrhea and were scored with 100 points. Ten percent of the farms (*n* = 3) had up to 4% of the animals with diarrhea, being scored with 70 points. Finally, 50% of the farms (*n* = 15) had more than 4% of animals with diarrhea, ranging from 8 to 20%, and were scored with 0 points. Ninety-seven percent of the farms (*n* = 29) had no animals with mange, so an excellent was obtained for this parameter, but in one farm 45% of the animals were affected with mange and 40 points were subtracted from the whole criterion for this farm. Sixty-three percent of the farms (*n* = 19) did the weaning when kits were 35 days of age or older, ranging from 35 to 37 days, and scored with an excellent, while the rest 37% were weaned between 31 and 33 days old, and 20 points were taken from the whole criterion for these farms. Seventy-three percent of the farms (*n* = 22) had neither flies nor fly eggs, so they were scored with an excellent, while the rest, 27%, had flies and remains of eggs, so 20 points were subtracted from the whole criterion for these farms. In total, 27% of the farms (*n* = 8) were classified as excellent for the criterion (scores ranging from 80 to 95 points). Fifty percent of the farms (*n* = 15) were classified as enhanced for the criterion (scores ranging from 57 to 79 points). Finally, 23% of the farms (*n* = 8) were classified as acceptable for the absence of diseases criterion (scores ranging from 40 to 47 points).

In relation to the criterion of absence of pain induced by management, only two of the farms performed the emergency killing with any of the methods considered in the protocol, and were scored with 100 points. The farmers not using any of these systems were using cervical dislocation for adults or blunt force on the head for very young animals, and were scored with 0 points. In addition, in one of the farms the animals were mutilated in the ears for identification. The rest were using tattoos. Overall, 6% of the farms (*n* = 2) were classified with an excellent, and the rest with not acceptable for this criterion.

When the score of the whole principle was considered, only one farm had an excellent for the good health principle (scored with 86 points). Sixty percent of the farms (*n* = 18) were classified with an enhanced for this principle, ranging from 56 to 78 points. Finally, 37% of the farms (*n* = 11) were classified as acceptable for the Good Health principle, ranging from 40 to 54 points.

### Appropriate Behavior

The Appropriate Behavior principle is assessed by means of the combination of three criteria: social behavior, other behaviors, and human-animal relationship.

In relation to the criterion of social behavior, no animals biting other animals were found in any of the farms assessed, so 100 points were obtained in all cases for this parameter. However, none of the animals were sufficiently protected from other adults due to the short distance between cages and the lack of a solid wall to protect the animals from being bitten, so 100 points to the whole criterion was subtracted in all cases. Consequently, all of the farms had 0 points and were classified as not acceptable for the social behavior criterion. However, no animals visually isolated from other animals were found in any farm.

In relation to the criterion of other behaviors, none of the animals in any of the farms assessed were found performing abnormal behaviors, so an excellent (100 points) was obtained for this parameter in all cases. On the other hand, none of the farms provided enrichment material to their animals, so 0 points were obtained for this parameter in all cases. In the farms where this could be assessed, does were provided with nesting material of good quality 24 h before kindling, so the excellent was always obtained in this parameter. In addition, the time of the day when the nest was open to the doe for suckling was every day at the same hour and with the same order in all farms assessed, so an excellent was obtained for this parameter. Globally, all farms were classified as enhanced for this criterion, with 60 points in total.

In relation to the human-animal relationship criterion and, more specifically, to the human approach test, the percentage of animals touching the stick was, in all cases, the reaction of the animal that defined the final score of the parameter due to the limited percentage of animals doing it. In fact, 20% of animals touching the stick was achieved only in one farm, and consequently scored with 100 points for this parameter. Thirty percent of the farms (*n* = 9) had at least 9% of the animals touching the stick, with percentages ranging from 10 to 18%. The rest of the farms, 67% (*n* = 20), were scored with 0 points, with percentages ranging from 0 to 8%. Thirty-three percent of the farms (*n* = 10) had all of the personnel in contact with the animals trained in specific courses on animal welfare, and they were scored with 100 points for this parameter. Another 10% of the farms (*n* = 3) did not have all the personnel, but at least one person, trained on animal welfare, and were scored with 50 points. The rest of the farms, 57% (*n* = 17) did not have any training for any of their personnel on animal welfare. In all farms, kits were touched once a day, so the excellent was obtained for this parameter. Globally, 7% of the farms (*n* = 2) were classified with an enhanced for this criterion (65 points). Forty-three percent of the farms (*n* = 13) were classified with an acceptable for this criterion (score ranging from 30 to 50 points), and 50% of the farms (*n* = 15) were classified as not acceptable for human-animal relationship (score ranging from 0 to 15 points).

When the score of the whole principle was considered, all of the farms were classified as acceptable for Appropriate Behavior, with values ranging from 21 to 41 points.

### Overall Assessment

Considering a global score for all of the farms, where Good Feeding provides 15%, Good Housing 30%, Good Health 35%, and Appropriate Behavior 20% of the final score, none of the farms was classified as excellent (Table 6). Eighty-three percent of the farms (*n* = 25) were classified as enhanced, with scores ranging from 56 to 74 points. Finally, 17% of the farms (*n* = 5) were classified as acceptable, with scores ranging from 41 to 54 points.

**Table 6:** Score obtained (from 0 to 100 points) for the different criteria (C1: Absence of prolonged hunger; C2: Absence of prolonged thirst; C3: Comfort around resting; C4: Thermal comfort; C5: Ease of movement; C6: Absence of injuries; C7: Absence of diseases; C8: Pain induced by management; C9: Social behavior; C10: Other behaviors; C11: Human-animal relationship), for the different principles (P1: Good feeding; P2: Good housing; P3: Good health; P4: Appropriate behavior) and the overall score as the result of the combination of the scores of the 4 principles (TOTAL) by farm (from 1 to 30).

**Farm**	**C1**	**C2**	**C3**	**C4**	**C5**	**C6**	**C7**	**C8**	**C9**	**C10**	**C11**	**P1**	**P2**	**P3**	**P4**	**Total**
1	100	75	35	0	100	55	82	0	0	60	0	91	49	55	21	52
2	100	65	80	100	75	60	67	0	0	60	0	88	83	51	21	60
3	100	85	65	0	75	55	75	0	0	60	35	95	52	52	32	54
4	100	65	80	100	75	90	64	0	0	60	35	88	83	62	32	66
5	100	65	80	100	75	100	67	0	0	60	0	88	83	67	21	66
6	100	75	80	100	15	100	40	0	0	60	30	91	62	56	30	58
7	100	65	80	100	100	100	95	0	0	60	35	88	92	78	32	74
8	100	75	80	100	100	45	72	0	0	60	35	91	92	47	32	64
9	100	90	65	0	100	100	92	0	0	60	0	97	61	77	21	64
10	100	75	80	0	90	100	85	0	0	60	35	91	64	74	32	65
11	100	85	80	0	90	55	57	100	0	60	50	95	64	65	36	64
12	100	75	80	100	40	60	47	0	0	60	0	91	71	43	21	54
13	100	85	80	0	90	100	57	0	0	60	35	95	64	63	32	62
14	100	85	80	100	100	70	72	0	0	60	0	95	92	57	21	66
15	100	75	72	100	20	70	47	0	0	60	65	91	61	47	41	56
16	100	75	60	0	90	40	60	0	0	60	0	91	56	40	21	48
17	100	75	80	100	45	70	74	0	0	60	0	91	73	58	21	60
18	100	75	80	100	15	85	47	0	0	60	30	91	62	53	30	57
19	100	85	80	0	90	85	90	0	0	60	0	95	64	70	21	62
20	100	75	72	100	85	60	47	0	0	60	65	91	84	43	41	62
21	100	65	35	0	40	70	40	0	0	60	0	86	28	44	21	41
22	100	85	60	100	100	70	79	0	0	60	30	88	84	60	30	65
23	100	85	80	100	75	100	91	0	0	60	30	95	83	76	30	72
24	100	85	80	100	75	70	80	0	0	60	0	95	83	60	21	64
25	100	65	60	100	100	70	79	0	0	60	30	88	84	60	30	65
26	100	85	80	0	55	100	47	0	0	60	50	95	51	59	36	57
27	100	85	80	100	60	100	65	100	0	60	15	95	78	86	26	73
28	100	85	80	100	100	85	62	0	0	60	15	95	92	59	26	67
29	100	85	52	100	75	100	80	0	0	60	0	95	72	72	21	65
30	85	85	60	100	100	40	71	0	0	60	30	85	84	44	30	59

## Discussion

The 30 farms assessed in the present study were all in the first year of integration in an animal welfare certification scheme, so the results could be biased in two ways. First, the producers know the protocol that will be used to assess their farms in advance and they enter the scheme voluntarily, so it is supposed that only those farmers really convinced of the capacities of their own farm to be approved would enter the scheme. Second, to be approved in this scheme a score of enhanced is needed in the global score, so only in cases of a very bad self-assessment of the farm is it expected to have global values below 55 points. Considering these two points, the objective of this discussion is to highlight the strengths and weaknesses of the farms assessed, the parameters used, and the protocol.

### Good Feeding

Within the Good Feeding principle, only one animal-based parameter is used: body condition score. According to the study of Bonanno et al. (14), the body condition score at artificial insemination (around 11 days post-partum) is a reliable indicator. However, later in the production cycle, when lactating does have simultaneous energy requirements for lactation and pregnancy, is as well a good moment for assessing body condition. For this reason, in the present protocol this parameter is assessed in animals around insemination and just before weaning. The way to assess this is based on Popescu et al. (15), where five categories were considered (emaciated, lean, ideal, fat, and obese), and simplified according to Welfare Quality (16), with just two categories, good (ideal) and lean (lean and emaciated). Popescu et al. (15) found more than 20% of the animals with problems of body condition in the two farms assessed, while in the present study 0% of animals affected in 30 farms was found. However, according to the authors, the low body-condition score found in their farms had, as the most probable cause, health-related reasons, and the present study had very good results for most of the parameters related to diseases. This, combined with the high replacement rates reported in some farms and the good hygienic conditions found, could explain these extremely good values in the body-condition score. Feeders should be positioned so that rabbits can easily access them while ensuring the feed is not contaminated by manure or urine. In 29 of 30 farms the cleanliness of feeders was excellent, so this seems to be under control in the farms assessed. According to NFACC (17), visually assessing kits for a milk spot in the first 5 days is a practical and effective way of confirming milk intake in kits. In the present study, all farmers carried out this practice for at least the first 7 days of life of the animals. In addition, all farmers were providing solid feed as well to the kits at 21 days of age. Kits gradually begin eating solid feed around 16–18 days of age, but usually their intake is in the form of milk until 25 days of age (17). Therefore, although the demand for providing solid feed could be advanced some days, it is not clear if this would really benefit the kit rabbits. Globally, this criterion is the one with the highest scores of the protocol. Although it could be argued that the absence of hunger and malnutrition is a basic principle for a professional rabbit producer, the low variability found could be caused by a low sensitivity of the parameters selected in detecting minor problems, so probably an effort is needed to include other parameters or to make the ones already included more sophisticated.

Rabbits have high water requirements and consume approximately twice as much water as feed, but there are no animal-based indicators available to measure prolonged thirst in a farm by visual inspection. Also, for other species, no animal-based parameters are currently in use for this criterion, and alternatively the number of drinking points and sometimes also the cleanliness and functioning of drinkers is assessed (16). Rabbits must have continuous access to safe, clean drinking water. In fact, it is important that nipples are clean, e.g., no hairs visible or mold, and working perfectly. In the farms assessed, in all cases except one, the drinkers were clean and all animals had at least one drinker working properly. In this criterion, the highest variability between farms was found in the height of the drinkers. The smallest weaned rabbits should also be able to drink, so the nipples should not be too high, an excellent height being when they are <14 cm from the floor, something found in 50% of the farms. Thus, although no animals could be considered thirsty during the assessment, this criterion (which could be called, as well, access to water) showed a higher variability between farms than did absence of prolonged hunger.

### Good Housing

Facility design and maintenance significantly impacts rabbit health and welfare. Housing systems need to provide a comfortable environment for rabbits through appropriate space allowance. Floor space impacts a rabbit's ability to thermoregulate in high ambient temperatures [rabbits can cool themselves by stretching out; (17)]. In fact, rabbits' use of space depends on ambient temperature and various characteristics of the enclosure [e.g., platform; (18)]. Therefore, in the Good Housing principle all of these aspects should be taken into account. Some animal-based measures related to comfort around resting are fully stretched, wet, and dirty animals. Most of the farms assessed had at least 20% of the animals fully stretched, and the maximum achieved was 65% of the animals with this posture, so the results for this parameter were quite good. In addition, the values for wet and dirty animals were excellent in most of the farms. In fact, wire-mesh flooring allows easy passage of manure and urine, is easily cleaned and sanitized, and is associated with lower rates of gastrointestinal disease and better air quality in commercial production systems (17). Nevertheless, certain types of wire-mesh flooring may increase the prevalence of pododermatitis in adult rabbits and the routine provision of a slatted plastic resting mat improves animal comfort and reduces the occurrence of this problem (17). In the present study, 90% of the farms were providing these resting mats to all their animals, which is a good sign concerning the quality of the farms assessed, and follows the progress already found by Rosell and de la Fuente (19), where their use in Spanish farms is described to have increased from 28% in 2001 to 75% in 2012. On the other hand, injuries inflicted on the kits by the doe can be minimized by enabling the doe to retreat from kits once they begin leaving the nest box, for instance by providing a platform (20). Platforms enable the possibility for more movement and can improve bone quality by enabling weight-bearing activity [e.g., jumping; (17)]. However, none of the farms provided these platforms to the animals. The farmers justified this because, unlike the provision of resting mats, the inclusion of platforms implies an enormous investment due to dramatic modifications needed in the cages and facilities. This could be an example of how a label system that could provide some extra added value to the final product could be used for investments to improve the welfare of animals, as this one could be an area to prioritize.

The respiratory tract of rabbits is irritated by fine dust in the air (21), so dust levels should not be too high. Nonetheless, there is no literature showing which dust levels are acceptable or not. For other species, Welfare Quality applies a dust sheet test which is a simple procedure indicating the amount of dust in the air (16), and applying the same methodology, in this study, 100% of the farms obtained an excellent rating for this parameter. In fact, the subjective assessment of the auditor agreed with this result, as the farms assessed were in all cases well-ventilated. Another aspect where all of the farms obtained an excellent was the quality of nesting material. Young kits, particularly those <2 weeks of age, have a very limited ability to thermoregulate, so properly bedded nest boxes provide warmth, minimizing chilling and mortality (17). Finally, light quality is another important aspect to consider when comfort around resting is considered. Lighting should provide uniform illumination and permit effective observation of rabbits. A light intensity of 30–50 lux at the rabbit level is necessary to enable mature rabbits to investigate their surroundings, have visual contact with other rabbits, and show active behaviors (11). Continuous lighting (i.e., no dark period in a 24-h cycle) negatively impacts welfare and health, and a natural light-dark pattern enables the rabbit to apply its natural rest-activity rhythm (17). Although light intensity and light-dark pattern was correct in 87% of the farms assessed, four farms failed in this parameter, all four of them for providing fewer than 8 h of enough light to the animals.

The second criterion to consider inside good housing is thermal comfort. When ambient temperatures exceed 25°C, rabbits begins to be at risk of heat stress, which may be indicated by decreased feed intake, increased water intake, open-mouthed panting with the head extended backwards, salivation, and ears fully upright and expanded with prominent blood vessels (21). When ambient temperatures exceed 35°C, rabbits can no longer regulate body temperature and are at significant risk of hyperthermia and heat stroke (22). However, the effective environmental temperature (i.e., the temperature that animals actually feel) may differ by several degrees from that measured in the overall barn and depends on several factors, such as air speed and temperature, relative humidity, flooring and cage/pen type, bedding, single or group housing, and the animal's stage of production and health status (17). As it is not possible to always register all of these variables, in the protocol a range is proposed of acceptable temperatures that can reach 28°C, but the score obtained can be corrected if the animals are seen panting. In addition, data records of maximum and minimum daily temperatures in the long-term are requested. None of the farms assessed had any animal showing panting or shivering, but one-third of the farms failed in the score because of the lack of data, so this is another important point for future improvements.

Ease of movement mainly considers height of the cage and space allowance. Space allowance affects a rabbit's ability to perform behaviors important to the species (e.g., grooming, hopping, jumping), and to adopt normal resting postures (ventral and lateral) and sitting postures [sitting upright or with all four legs on the ground; (17, 18)]. Providing an area within the cage/pen with a minimum height of 40 cm promotes the expression of natural behavior and reduces the risk of ear lesions (17). In the present study, only one farm had a height of 40 cm, and only 30% reached 38 cm. In terms of space allowance, a breeding rabbit toward the end of pregnancy (4 kg−5 kg live weight) would need a cage with a minimum of 3,500 cm^2^ according to the EFSA (11), but only 50% of the farms in the present study had at least these 3,500 cm^2^, with some farms having 4,000 cm^2^. Although Mirabito et al. (23) did not observe any significant difference in reproductive time or budget time of reproducing does kept in cages with different available surfaces (about 3,400, 4,500, and 5,900 cm^2^), Dresher (24) showed a great reduction in abnormal skeletal developments in cages of 3,500 cm^2^ as compared to 2,400 cm^2^. Therefore, a strategy, probably again with a label giving an added value to pay for the investment necessary, is needed to encourage all farms to be able to arrive to at least the 3,500 cm^2^ and 40 cm of height.

### Good Health

This principle includes absence of injuries, diseases, and pain induced by management. Rabbits should not have any skin damage or wounds. Wounds can be caused by inadequate equipment (e.g., sharp parts of cages), or by mutilative or aggressive behavior of other rabbits. In general, the results obtained in the present study were very positive in this respect, with only a few farms (*n* = 4) with some animals with wounds on the body, and none with problems in the ears or gait score. Pododermatitis should be considered apart. The condition begins with localized hair loss and callous formation on the footpad and the hind feet. It progresses to cracked and open calluses and is most severe when open wounds or ulcers have formed (17). Although 90% of the farms had rest mats, only 48% of the farms obtained an excellent for this parameter, and 30% of the farms had more than 8% of the animals with severe cases, so other strategies should be considered to reduce the incidence of this painful problem. Routine maintenance of facilities and timely replacement of cages/pens before their condition deteriorates helps prevent rabbits from becoming injured, but at least two cages in 27% of the farms assessed were considered dangerous for the animals, so this is an important point to consider, and this justifies that 30% of the total score of the criterion could depend on this fact independently of the values found in other animal-based parameters.

To check if rabbits suffer from disease, they can be checked for a number of clinical signs that are indicative of health problems, like coughing and sneezing, nasal and ocular discharge (25); mange, dermatitis (26), or diarrhea (25). In the present study, coughing, sneezing, ocular discharge, and neck torsion were not found. In addition, just one farm had animals affected by skin abnormalities, such as dermatitis, another one with animals affected by enteropathy, and another one with problems of mange. In fact, the two health problems most seen were nasal discharge, with 17% of the farms with more than 4% of the animals affected (and a maximum of 12%), and diarrhea, the most predominant problem, with 50% of the farms with more than 4% of the animals affected (and a maximum of 20%). However, globally, the presence of diseases in the assessed farms could be considered low. On the other hand, mortality is an important indicator of herd health to monitor on a farm (17, 27, 28). In fact, in breeding rabbits, mortality is often due to infectious causes (17), and a reduction in this indicator represents an improvement in animal health (28). When monthly mortality in breeding does and bucks due to adverse health issues and injury exceeds 5%, it should be considered as an alarm signal (17). In this respect, 50% of the farms assessed had mortalities in the last 3 months below 5%, being considered acceptable values, and 17% of the farms had <3% of mortality, being considered excellent values. Therefore, globally, the values of mortality confirmed the general good health found the day of the visit. Nevertheless, another 17% did not have data on mortality. This should be considered unacceptable and leave the farmer who is not providing these numbers out of any certification system. In any case, mortality records should be interpreted in conjunction with culling records. In fact, for all conditions affecting rabbit health and welfare, early recognition and prompt treatment or euthanasia are essential to minimize animal pain and distress (17). Thus, in the present protocol, the percentage of culling is also assessed, and it is considered that, ideally, the animals culled in a farm should be more than those deaths without human intervention, the total numbers being, of course, as low as possible. According to Rosell and de la Fuente (29), the median monthly removal risk in does in Spain from 2000 to 2005 was 9.3%, with 3.4% dead and 5.7% culled. Therefore, in a period of 3 months, as assessed in the present protocol, numbers would be around 10% of mortality + 15% culling, so most of the farms assessed in the present study present a clear improvement in these numbers (Figure 2). However, 40% of the farms had higher mortality rates than culling rates, so emergency killing is still something to be improved in an important number of farms. For does, and related to culling, the percentage of replacement may be a good indicator of health. According to Marai et al. (30), percentage of replacement of does varies between 70 and 160% per year. In the present study, 13% of the farms ranged from 35 to 70%, and all of them showed an excellent result in relation to the presence of diseases. In fact, two of them, Farms 7 and 27, obtained the highest overall scores of the 30 farms, 74 and 73 points, respectively. Nonetheless, to have good values in this parameter is not a guarantee of high final scores, as Farm 3, with a replacement rate of 35%, obtained a final score of 54 points (Table 6), so one point below the objective of the 55 points. In this case, a lower score for the Good Housing principle in comparison to other farms would be the cause. In fact, it was one of the only three farms where rest mats were not present for 100% of the animals the day of the visit (with 8% of animals with severe pododermatitis) and, as in other farms, no data were taken for temperatures. Therefore, this is a good example of how animal welfare assessment should comprise a combination of different measures instead of a single indicator. The fact that 70% of the farms had a replacement of more than 110% is related, as well, to the high rhythms of intensification of most of the rabbit farms. Actually, only 13% of the farms leave at least 49 days between parturitions in does, with most of the farms leaving just 42 days. This is another point that could be improved with a label giving added value to the product.

While milk production varies between does, daily milk production typically peaks toward the end of the third week of lactation and then drops rapidly (21), coinciding with the period in which kits' intake of solid feed increases. In natural conditions, if the doe is not pregnant, litter weaning is completed within the fifth and sixth weeks of age (31). This is also an important time to consider welfare in rabbit production, as it has been shown there is an association between later weaning and increased risk of enteritis because of increased stress (17). In fact, long weaning times affect the welfare of the doe due to an increased demand of energy when lactation is combined with gestation (especially in short intervals between parturitions) and the impossibility to escape from offspring in most usual facilities. Therefore, although the objective of the present protocol is to give the highest score to the farms weaning the animals at 35 days of age or later, and this occurred in 63% of the farms, the objective of the protocol should be to balance this parameter with long intervals between parturitions and improved facilities to provide some opportunities to the doe for escaping from the kits, all combined with lower replacement rates and good general health status. Finally, another aspect to consider in the absence of disease criterion is the general cleanliness of the facilities, scored with an excellent in 100% of the farms assessed. Related to cleanliness, rodents and insects are recognized as carriers of many diseases. As the control of rodents should be a legal requirement, the protocol is centered on the presence of flies. In this respect, 73% of the farms had neither flies nor fly eggs present in the farm, while the rest had both flies and their eggs present, being considered a risk for diseases and consequently penalized in this criterion.

Pain induced by management includes two main aspects, the mutilations performed on the animals for identification and how emergency killing or euthanasia is performed. Euthanasia is defined as the “ending of the life of an individual animal in a way that minimizes or eliminates pain and distress” (32). It is characterized by rapid, irreversible unconsciousness followed by prompt death (33). Euthanasia is an important aspect of animal welfare. Allowing a sick or injured rabbit to linger unnecessarily is unacceptable. Any euthanasia method must result in rapid loss of consciousness followed by death without the animal regaining consciousness (33). Neck dislocation was the system used by 93% of the farmers for does and bucks in the present study. According to the recommendations of EFSA (34), cervical dislocation is considered a killing method and therefore it should only be applied on unconscious animals. In addition, the hazards related to cervical dislocation include “manual restraint” (leading to pain and fear) and “incorrect application” [leading to the absence of unconsciousness, pain, fear, and distress; (34)]. For these reasons, in the present protocol, the system is not considered as correct. Certainly, this is one critical point that needs to be improved in the rabbit farms assessed. For identification, ear-marks (metal or plastic), microchips, or tattoos can be used. There are countries where ear-marks are not allowed, for being potentially painful to the rabbit (17) or causing injury by being caught on the cage wire. Consequently, in the protocol it is asked to not use this system, which agrees with the practice of 97% of the farms assessed, which use tattoos for identification. However, Keating et al. (35) described an acutely painful procedure for rabbits related to tattooing the ears and suggest the use of anesthesia to mitigate the associated pain. Therefore, to ask for the use of anesthesia, if tattooing is needed for identification, could be an appropriate refinement of the protocol.

### Appropriate Behavior

Managing territoriality and associated aggression in pair- or group-housed does is difficult. Rates of doe injuries and kit mortality are typically higher in pair and group systems (17). This was not a problem in the farms assessed in the present study, as all adult animals were housed in single cages. However, when cages are too close to each other, dominant animals can try to bite the adjacent subordinates when lying by the cage wall. This would have a higher risk of injuries, prevents a correct resting behavior in the subordinate animal, and induces a higher alert level in the dominant one, so it is suggested to have a good separation between cages or provide solid walls to prevent contact between individually housed rabbits (36). In the present study, although no animals were found biting other animals, cages did not have solid walls and the distance between cages was insufficient, so 0 points were obtained in all cases for the social behavior criterion. However, a recent paper (37) describes that adult rabbits are better in pairs than alone. Although this study is done in neutered rabbits in outdoor conditions with low temperatures where huddling was needed to maintain body temperature and in just 45 individuals from a rabbit-only rescue center, this should be considered in further studies.

Both in growing and adult animals, stereotypes, which is abnormal behavior repeated obsessively without apparent aim, have often been described (38, 39). Stereotypes and abnormal behavior are indicators of reduced welfare in rabbits. These behaviors can be head shaking, swaying, wire gnawing, wall pawing, and over-grooming (11, 40–42). In the present study, none of the animals were observed showing these behaviors. Although the methodology, 2 min of observation per animal, was tested previously in other farms with positive results (presence of stereotypes) and the time dedicated to assess abnormal behaviors is double that used in other species, such as pigs, for the same purpose (16), it cannot be discarded that some adjustment could be necessary to the methodology to increase its sensitivity. In any case, rabbits perform fewer abnormal behaviors (e.g., oral stereotypies, cage biting, or manipulation) when provided with enrichment material (17), so this is another important point to consider in the protocol. Examples of enrichment are hard wood-gnawing of blocks or sticks, hay, straw or litter (for chewing or manipulation), grass or hay in any form, tubes/tunnels, and mirrors (17). Nevertheless, none of the farms assessed was using any kind of enrichment for their rabbits, so this is clearly another gap that the rabbit producers need to address. An important exception to the absence of any type of enrichment material is the provision of nesting material for does 24 h prior to kindling. If insufficient nesting material is present, the doe cannot perform her natural nest building behavior (43). A variety of bedding materials may be used, including rabbit hair, hay, straw, shredded paper, and wood shavings, but in any case, it should be dry and dust free. In this case, nesting material for the doe was found to be proper (in quantity and quality) in all farms assessed.

In natural conditions, after kindling and attending to the newborn kits, the doe leaves the nest, closes it up, and comes back only to suckle the kits. According to Trocino and Xiccato (31), suckling takes place once a day, usually after sunset, and lasts a few minutes (two to five), during which the kits ingest a high quantity of nutritive substance and energy enough for rapid development and growth. However, Hoy et al. (44) described that only 56% of the does free to enter cages at will, in fact, nursed their kits only once a day, whereas 40% nursed twice or more often during the day and 4% did not nurse at all. Most of the rabbit farmers, and 100% of the ones assessed in this study, reproduce this behavior in their farms by means of a controlled lactation. This is to open the nest for allowing the doe to visit her kits just once a day and for a few minutes. The advantages of controlled lactation are a reduction in kit mortality due to crushing and higher kit weight homogeneity (45). In addition, it helps to confirm that nursing is occurring. Further, not keeping to a regular timetable and leaving the doe waiting to access the nest negatively compromises her welfare through increased anxiety and likely physical discomfort caused by delayed nursing opportunity. For this reason, the protocol considers at which hour this operation is to be carried out every day or whether the nest is freely accessible to the doe throughout the 24 h. In addition, it might be appropriate to consider whether providing access to the nest twice a day should score higher than once a day. The few differences among farms, especially because of the absence of abnormal behaviors and the absence of enrichment material, produced a high homogeneity in the final score of the whole criterion, with 60 points in all cases.

A good human-animal relationship promotes rabbit welfare. With proper handling, rabbits experience less stress and fear, and the risk of injury to the animals and handlers is greatly reduced (11). In other species, the human-animal relationship is assessed by means of an approach test (16). The approach test of the present protocol is based on those proposed by Hansen and Moller (46) for minks maintained in cages but also considering the lack of aggressivity of rabbits against the stick. The range of animals touching the stick was from 0% in the worst farm to 20% in the best one. Although 30 farms are not probably enough to have a complete picture of the situation, it is possible that in the future the thresholds of what is acceptable and excellent could be adjusted to lower percentages to increase the capacity of discrimination of the parameter. Another important point to consider in this criterion is training. Management practices have a significant impact on animal health, welfare, and productivity (47). In addition, training and knowledge development about rabbit welfare and care should be an ongoing process. Nevertheless, 57% of the farms assessed did not have any person trained in animal welfare, so this is again a critical point that producer associations should try to solve. Among the actions that may be adopted to improve human-animal relationships, early manipulation has been shown to provide positive results, especially if it is applied during a sensitive period in the first week post-partum and near the time of nursing, due to a general increase in arousal that occurs at this time (48). For this reason, it is asked in the protocol if the kits are touched at least once a day during the first week of age. All of the farms assessed were performing a controlled lactation and ensuring that all kits were taking in milk (see Good Feeding, above) by holding the animals gently and checking the abdomen, so an excellent was obtained for this parameter. Globally, this was the criterion with the most variability within the three of the Appropriate Behavior principle.

### Global Assessment

As commented previously, the farms assessed in the present study were asked to obtain a minimum of 55 points in the global score and they had the opportunity to check the assessment protocol prior to being audited under a voluntary basis. Therefore, few farms, even none, should be expected to score below 55 points. However, this occurred in five farms. Three of them were actually very close to 55 points (52 and 54 points) and the other two not (41 and 48 points). In addition, 6 other farms were between 55 and 60 points (Table 6). In all of these cases, it is encouraged to carefully review the critical points found during the assessment to improve the final score. In the four farms below 55 points this is mandatory, but for the other farms it can be important as well for not having problems in the future. In fact, as mentioned previously, the disease results were fine, in general, in the assessed farms, so these farms so close to 55 points are at risk if, in the next assessment, the health score, due to punctual problems, has a worse result. In addition, the rest of the farms are still far from the 100 points or even the excellent, due to not dealing with health issues punctually in order to reduce suffering. So in all cases there are opportunities for improvement. Therefore, the protocol can be used as well as a tool for identifying gaps and planning future investments. Good Housing and Appropriate Behavior are the two principles with the lowest weights in the final score (15 and 20%, respectively), and at the same time they are the principles with the lowest variability. In the first case it is because of a very high score, and in the second because of a very low score. Although a future version of the protocol could try to add more variability to the first principle and penalize in the final value more if the last one has such low scores, the results just show how, for years, rabbit producers have been very focused on feeding needs and very few on behavioral needs.

## Conclusions

In general, most of the farms obtained a good overall score, the maximum found being 73 points. Nevertheless, none of the farms obtained an excellent, and four farms were scored below the 55 points required. The Good Feeding principle obtained the highest score, reaching an excellent in all farms, and Appropriate Behavior the lowest one, with values ranging from 21 to 41 points out of 100. In general, the main problems found were absence of platforms, low space allowance and height of the cage, inappropriate system for performing emergency killing, insufficient protection of does from other adjacent does when housed individually, absence of enrichment material, and, in some cases, the lack of temperature data records, high replacement rates, and even lack of mortality rate data records.

## Data Availability Statement

The raw data supporting the conclusions of this article will be made available by the authors, but maintaining blinded the farms of origin.

## Ethics Statement

Ethical review and approval was not required for the animal study because the study is focused in the assessment of farm animal welfare in commercial farms, so no interventions of any kind were carried out on the animals.

## Author Contributions

AD was responsible for the development of the protocol, collecting the data, writing the paper, and training the assessor in using the protocol. JP and XM were collaborators in developing the protocol and testing it in commercial farms during its development to refine the parameters. All authors contributed to the article and approved the submitted version.

## Conflict of Interest

The data was obtained from farms interested in being certified on animal welfare within a certification scheme owned by IRTA. Therefore, although it exists as a commercial relationship, the farmers were just subjected to the audit with no possibilities of any other intervention in the study. The authors therefore declare that the research was conducted in the absence of any commercial or financial relationships that could be construed as a potential conflict of interest.
