# Supplementary material for: Animal Welfare Assessment Protocol for Does, Bucks, and Kit Rabbits Reared for Production
Source: Front Vet Sci. 2020 Aug 7;7:445. doi: 10.3389/fvets.2020.00445 (PMC7438856; doi:10.3389/fvets.2020.00445)
Supplement: Supplementary file 1 [file Table_1.DOCX]

Supplementary Table 1. Example of an imaginary farm with some of the possible results for the different measures in the column named “result” and the resulting calculations according to the score given to each measure (Score), the weight of the measure inside the criterion (Weight-1), the score obtained for the criterion (Sum-1), the weight of the criterion inside the principle (Weight-2), the score obtained for the principle (Sum-2), the weight of the principle in the overall score (Weight-3) and the final result for the imaginary farm (Sum-3). The scores for criteria, principles and overall are shown in colours, where blue is excellent: from 80 to 100 points; green enhanced: from 55 to 79 points; orange acceptable: from 20 to 55 points and red not acceptable: less than 20 points.

| Result | Score | Weight-1 | Sum-1 | Weight-2 | Sum-2 | Weight-3 | Sum-3 |
| --- | --- | --- | --- | --- | --- | --- | --- |
| 4% of lean animals | 0 points | Multiply x 0.70 | 0 |  |  |  |  |
| 100% of clean feeders | 100 points | Multiply x 0.15 | 15 |  |  |  |  |
| All kits checked after doe’s visit | 100 points | Multiply x 0.15 | 15 |  |  |  |  |
| Access to solid food in kits is ok | -0 points | 0 | 0 |  |  |  |  |
| Total score for absence of prolonged hunger criterion | | | 30 | x 0.65 | 19.5 |  |  |
| Just 1 drinker per animal | 65 points | Multiply x 0.45 | 29.25 |  |  |  |  |
| 100% functioning | 100 points | Multiply x 0.35 | 35 |  |  |  |  |
| 98% clean | 50 points | Multiply x 0.25 | 12.5 |  |  |  |  |
| Height of the drinker at 23 cm | -20 points | -20 | -20 |  |  |  |  |
| Total score for absence of prolonged thirst criterion | | | 57 | x 0.35 | 19.95 |  |  |
| Total score for the Good Feeding principle | | | | | 39.45 | x 0.15 | 5.92 |
| 30% of fully stretched animals | 100 points | Multiply x 0.15 | 15 |  |  |  |  |
| 7% of wet animals | 60 points | Multiply x 0.20 | 12 |  |  |  |  |
| 6% of animals moderately dirty | 0 points | Multiply x 0.20 | 0 |  |  |  |  |
| No dust presence | 100 points | Multiply x 0.15 | 15 |  |  |  |  |
| 55% of animals with resting mats | 50 points | Multiply x 0.30 | 15 |  |  |  |  |
| No presence of platforms | -20 points | -20 | -20 |  |  |  |  |
| Good light quality | -0 points | 0 | 0 |  |  |  |  |
| Clean and dry litter in all nests | -0 points | 0 | 0 |  |  |  |  |
| Total score for comfort around resting criterion | | | 37 | x 0.40 | 14.8 |  |  |
| Correct temperatures | 100 points | Multiply x 1.0 | 100 |  |  |  |  |
| Ok when burning hair | -0 points | -0 | -0 |  |  |  |  |
| 3% of animals panting | -50 | -50 | -50 |  |  |  |  |
| 0% of animals shivering | -0 points | -0 | -0 |  |  |  |  |
| Total score for thermal comfort criterion | | | 50 | x 0.25 | 12.5 |  |  |
| 98% with free movement | 65 points | Multiply x 0.30 | 19.5 |  |  |  |  |
| Cage height 30 cm in >10% | 0 points | Multiply x 0.30 | 0 |  |  |  |  |
| 3200 cm^2^/animal in all cages | 60 points | Multiply x 0.40 | 24 |  |  |  |  |
| Total score for ease of movement criterion | | | 43.5 | x 0.35 | 15.23 |  |  |
| Total score for the Good Housing principle | | | | | 42.53 | x 0.30 | 12.76 |
| 7% with wounds on the body | 0 points | Multiply x 0.25 | 0 |  |  |  |  |
| 0% with wounds on the ears | 100 points | Multiply x 0.15 | 15 |  |  |  |  |
| 3% with fallen ears | 70 points | Multiply x 0.10 | 7 |  |  |  |  |
| 9% with severe pododermatitis | 0 points | Multiply x 0.30 | 0 |  |  |  |  |
| 0 with gait score problems | 100 points | Multiply x 0.20 | 20 |  |  |  |  |
| 0 with hairless areas | -0 points | -0 | -0 |  |  |  |  |
| 1 cage with risk of injuries | -15 points | -15 | -15 |  |  |  |  |
| Total score for absence of injuries criterion | | | 27 | x 0.40 | 10.8 |  |  |
| 5 % of mortality | 70 points | Multiply x 0.10 | 7 |  |  |  |  |
| 2% of culling | 0 points | Multiply x 0.05 | 0 |  |  |  |  |
| 110% of replacement per year | 0 points | Multiply x 0.05 | 0 |  |  |  |  |
| 43 days between parturitions | 40 points | Multiply x 0.05 | 2 |  |  |  |  |
| 4% of animals coughing | 0 points | Multiply x 0.10 | 0 |  |  |  |  |
| 0% of animals sneezing | 100 points | Multiply x 0.10 | 10 |  |  |  |  |
| 5% with nasal discharge | 0 points | Multiply x 0.07 | 0 |  |  |  |  |
| 7% with ocular discharge | 0 points | Multiply x 0.08 | 0 |  |  |  |  |
| 0% with skin problems | 100 points | Multiply x 0.10 | 0 |  |  |  |  |
| 0% with neck torsion | 100 points | Multiply x 0.10 | 0 |  |  |  |  |
| 2% with enteropathy | 70 points | Multiply x 0.10 | 7 |  |  |  |  |
| 5 % with diarrhoea | 0 points | Multiply x 0.10 | 0 |  |  |  |  |
| No animals with mange | -0 points | -0 | -0 |  |  |  |  |
| 3 cages partly dirty | -10 points | -10 | -10 |  |  |  |  |
| Age of weaning correct (36 days) | -0 points | -0 | -0 |  |  |  |  |
| No flies either eggs present | -0 points | -0 | -0 |  |  |  |  |
| Total score for absence of diseases criterion | | | 16 | x 0.40 | 6.4 |  |  |
| Not correct killing method used | 0 points | Multiply x 100 | 0 |  |  |  |  |
| Mutilations for identification used | -20 points | -20 | -20 |  |  |  |  |
| Total score for pain induced by management criterion | | | 0 | x 0.20 | 0 |  |  |
| Total score for the Good Health Principle | | | | | 17.2 | x 0.35 | 6.02 |
| No animals biting others | 100 points | Multiply x 100 | 100 |  |  |  |  |
| No problems of isolation | -0 points | -0 | -0 |  |  |  |  |
| Total score for the social behaviour criterion | | | 100 | x 0.35 | 35 |  |  |
| 2% with abnormal behaviours | 55 points | Multiply x 0.60 | 33 |  |  |  |  |
| No enrichment material | 0 points | Multiply x 0.40 | 0 |  |  |  |  |
| Nesting material in all cases | -0 points | -0 | -0 |  |  |  |  |
| Time to access the nest >1h <2h | -10 points | -10 | -10 |  |  |  |  |
| Total score for other behaviours criterion | | | 23 | x 0.35 | 8.05 |  |  |
| 12% of animals touch the stick | 50 points | Multiply x 0.70 | 35 |  |  |  |  |
| Only 1 person trained on AW | 50 points | Multiply x 0.30 | 15 |  |  |  |  |
| Not all kits are touched | -10 points | -10 | -10 |  |  |  |  |
| Total score for the human-animal relationship criterion | | | 40 | x 0.30 | 12 |  |  |
| Total score for the Appropriate Behaviour principle | | | | | 55.05 | x 0.20 | 11.01 |
| GLOBAL SCORE FOR THE FARM (FROM 1 TO 100 POINTS) | | | | | | | 35.7 |
