# Supplementary material for: Animal Welfare Assessment Protocol for Does, Bucks, and Kit Rabbits Reared for Production
Source: Front Vet Sci. 2020 Aug 7;7:445. doi: 10.3389/fvets.2020.00445 (PMC7438856; doi:10.3389/fvets.2020.00445)
Supplement: Supplementary file 2 [file Table_2.DOCX]

Supplementary Table 2. Individual weight of each measure, criterion and principle in relation to the overall score of the protocol fixed in a maximum of 100 points.

| Item | Weight out of 100 points |
| --- | --- |
| Good Feeding principle | 15 points |
| Prolonged hunger criterion | 9.75 points |
| Body condition | 6.825 points |
| Cleanliness of feeders | 1.4625 points |
| Cleanliness of feeders | 1.4625 points |
| Access to solid food in kits | -1.95 points |
| Prolonged thirst criterion | 5.25 points |
| Drinking points per doe/buck | 2.3625 points |
| Functioning of drinkers | 1.8375 points |
| Cleanliness of drinkers | 1.3125 points |
| Height of drinkers | -1.05 points |
| Good Housing principle | 30 points |
| Comfort around resting | 12 points |
| Fully stretched animals | 1.8 points |
| Wet animals | 2.4 points |
| Dirty animals | 2.4 points |
| Dust | 1.8 points |
| Presence of Resting mat | 3.6 points |
| Presence of an elevated platform | -1.8 points |
| Light quality | -1.8 points |
| Quality of littered floor | -1.8 points |
| Thermal comfort | 7.5 points |
| Temperature | 7.5 points |
| Burning hair | -1.5 points |
| Panting | -7.5 points |
| Shivering | -7.5 points |
| Ease of movement | 10.5 points |
| Free movement | 3.15 points |
| Height of the cage | 3.15 points |
| Stocking density | 4.2 points |
| Good Health principle | 35 points |
| Absence of injuries | 14 points |
| Wounds on the body | 3.5 points |
| Wounds on the ears | 2.1 points |
| Fallen ears | 1.4 points |
| Pododermatitis | 4.2 points |
| Gait score | 2.8 points |
| Hairless areas | -1.4 points |
| Risk of injuries | -4.2 points |
| Absence of diseases | 14 points |
| Mortality | 1.4 points |
| Culling | 0.7 points |
| Replacement | 0.7 points |
| Time between parturitions | 0.7 points |
| Coughing | 1.4 points |
| Sneezing | 1.4 points |
| Nasal discharge | 1.0 points |
| Ocular discharge | 1.1 points |
| Dermatophytosis, dermatitis, abscesses | 1.4 points |
| Neck torsion | 1.4 points |
| Enteropathy | 1.4 points |
| Diarrhoea | 1.4 points |
| Mange | -5.6 points |
| Cleanliness of facilities | -2.8 points |
| Age of weaning | -2.8 points |
| Flies presence | -2.8 points |
| Pain induced by management | 7 points |
| Killing methods | 7 points |
| Mutilations for identification | 1.4 points |
| Appropriate Behaviour principle | 20 points |
| Social behaviour | 7 points |
| Negative social behaviour | 7 points |
| Isolated animals | -7 points |
| Other behaviours | 7 points |
| Abnormal behaviours | 4.2 points |
| Enrichment material | 2.8 points |
| Nesting material | -1.4 points |
| Time to access to the nest | -1.4 points |
| Human-animal relationship | 6 points |
| Human approach test | 4.2 points |
| Training of personnel | 1.8 points |
| Touching the kits | -0.6 points |
